# Substance P aggravates ligature-induced periodontitis in mice

**DOI:** 10.3389/fimmu.2023.1099017

**Published:** 2023-04-14

**Authors:** Yasir Dilshad Siddiqui, Xuguang Nie, Sheng Wang, Yasaman Abbasi, Lauren Park, Xiaoxuan Fan, Vivek Thumbigere-Math, Man-Kyo Chung

**Affiliations:** ^1^ Program in Neuroscience, Center to Advance Chronic Pain Research, Department of Neural and Pain Sciences, School of Dentistry, University of Maryland, Baltimore, MD, United States; ^2^ Department of Preventive Dentistry, College of Dentistry, Jouf University, Sakaka, Saudi Arabia; ^3^ Department of Microbiology and Immunology, Flow Cytometry Shared Service, University of Maryland School of Medicine, Baltimore, MD, United States; ^4^ Department of Advanced Oral Sciences and Therapeutics, University of Maryland School of Dentistry, Baltimore, MD, United States

**Keywords:** *Tac1*, substance P, periodontitis, mouse model, osteoclasts, cytokines, neutrophils

## Abstract

Periodontitis is one of the most common oral diseases in humans, affecting over 40% of adult Americans. Pain-sensing nerves, or nociceptors, sense local environmental changes and often contain neuropeptides. Recent studies have suggested that nociceptors magnify host response and regulate bone loss in the periodontium. A subset of nociceptors projected to periodontium contains neuropeptides, such as calcitonin gene-related peptide (CGRP) or substance P (SP). However, the specific roles of neuropeptides from nociceptive neural terminals in periodontitis remain to be determined. In this study, we investigated the roles of neuropeptides on host responses and bone loss in ligature-induced periodontitis. Deletion of tachykinin precursor 1 (*Tac1*), a gene that encodes SP, or treatment of gingiva with SP antagonist significantly reduced bone loss in ligature-induced periodontitis, whereas deletion of calcitonin related polypeptide alpha (*Calca*), a gene that encodes CGRP, showed a marginal role on bone loss. Ligature-induced recruitment of leukocytes, including neutrophils, and increase in cytokines leading to bone loss in periodontium was significantly less in *Tac1* knockout mice. Furthermore, intra-gingival injection of SP, but not neurokinin A, induced a vigorous inflammatory response and osteoclast activation in alveolar bone and facilitated bone loss in ligature-induced periodontitis. Altogether, our data suggest that SP plays significant roles in regulating host responses and bone resorption in ligature-induced periodontitis.

## 1 Introduction

Periodontitis is a common oral disease affecting over 40% of the adult population in the United States (1). It is primarily due to the result of microbial dysbiosis and the dysregulation of host inflammatory responses (2, 3). Persistent and uncontrolled inflammation in periodontitis leads to a progressive loss of periodontal tissues, including the alveolar bones that support the teeth. Alveolar bone is maintained by constant mechanical stimulation from the tooth and undergoes vigorous remodeling throughout life. Alveolar bone disruption that occurs in periodontitis is irreversible and is the primary cause of tooth loss.

The host inflammatory response to periodontal infection is regulated by a number of factors, including sensory neurons that innervate the periodontal tissues (4, 5). These sensory neurons transduce various noxious mechanical, thermal, and chemical stimuli. Among these, nociceptive afferents expressing transient receptor potential cation channel subfamily V member 1 (TRPV1), are largely peptidergic afferents that secrete a variety of neuropeptides to regulate the inflammatory process (6). Substance P (SP) and calcitonin gene-related peptide (CGRP) are the most abundant neuropeptides. Recent studies have suggested that nociceptors play a regulatory role in periodontitis (4, 5, 7). We have demonstrated that the activity of nociceptors exaggerates inflammatory tissue responses and facilitates bone loss in a mouse periodontitis model (4). Infection with pathogenic microbes leads to the activation of TRPV1 and transient receptor potential cation channel subfamily A member 1 (TRPA1), which are enriched in the peptidergic nerve terminals, and mediate the Ca^2+^-dependent release of neuropeptides causing neurogenic inflammation (8–10). SP and CGRP are implicated in multiple biological processes including inflammation (11, 12); however, the specific role of these neuropeptides from these nociceptive terminals in periodontitis remains to be determined.

SP was identified as an early marker for gingival inflammation in experimental periodontitis in humans (13). SP has been detected at significantly higher levels in the gingival crevicular fluid of patients with periodontitis than normal, and its level decreases after effective treatment (14–17). In contrast, CGRP is present in human gingival crevicular fluid at lower levels at sites of periodontitis than at healthy sites (18–20). Given that CGRP and SP are known to affect bone remodeling, they may serve as neurogenic factors that contribute to the regulation of host responses and alveolar bone loss in the periodontium. Therefore, in this study, we used genetically engineered mouse models to investigate the roles of neuropeptides in ligature-induced periodontitis.

## 2 Materials and methods

### 2.1 Experimental mouse models

All animal procedures were performed in accordance with the NIH Guide for the Care and Use of Laboratory Animals (Publication 85–23, Revised 1996) and were performed according to the University of Maryland-approved Institutional Animal Care and Use Committee protocols and the ARRIVE guidelines. C57BL/6 mice, *Tac1* ^−/−^ (Jax, 004103), and *Calca* ^Cre/Cre^ mice (Jax, 033168) were obtained from the Jackson Laboratory. *Calca^Cre^* is a knock-in/knock-out line in which Cre is targeted to the first coding exon of the mouse *Calca* gene, and therefore homozygote knock-in mice do not express *Calca.* Both *Tac1* ^−/−^ and *Calca^Cre/Cre^* mice procreate normally. Eight-week-old male and female mice were used in each experimental group. In all assays, the animals were randomly allocated to the experimental groups. Animals were group-housed under standard conditions with ad libitum access to water and food. The experimenters were blinded for the treatment groups during the analysis of the data in each assay.

### 2.2 Ligature-induced periodontitis

Mice were anesthetized using ketamine/xylazine (100-150 mg/kg of ketamine and 10-20 mg/kg of xylazine). A 5-0 silk ligature was placed around the second maxillary molar (M2), which remained in place in all mice throughout the experimental period. The suture was tied gently to prevent damage to the periodontal tissue. The ligatures remained in place in all mice throughout the indicated experimental period.

### 2.3 Micro-focus computed tomography

The animals were anesthetized with ketamine/xylazine and euthanized *via* transcardial perfusion using 4% PFA (4). Maxillae were hemisected and micro-focus computed tomography (µCT) images were obtained using a Siemens Inveon Micro-PET/SPECT/CT (Siemens, Ann Arbor, MI) at a 9 µm spatial resolution. Siemens Inveon Research Workplace 4.2 software was used for image acquisition and processing, 2-D and 3-D image viewing, and quantitative analysis. Unless otherwise indicated, bone loss was evaluated from the buccal side. To assess the levels of periodontal bone loss, four linear measurements were taken following 3-D reconstruction of the µCT scans. Bone loss was evaluated on the distal side of the distobuccal root of the first molar (M1), the mesial side of the mesiobuccal root of the second molar (M2), the distal side of the distobuccal root of M2, and the distal side of the distobuccal root of the third molar (M3). Unless otherwise indicated, distances from the cement-enamel junction (CEJ) to the most apical site of bone destruction were measured. Four measurements were averaged to obtain the distance from the CEJ.

### 2.4 Tissue processing and immunohistochemistry

Immunohistochemical assays of the trigeminal ganglion (TG) and maxillae were performed as previously described (4, 21–23). Maxillae were decalcified in 0.5M Ethylenediaminetetraacetic acid (EDTA) in PBS (pH 7.4) over the course of 14 days. Tissue processing was performed at the Histology Core at the University of Maryland School of Medicine for paraffin embedding and the tissues were sectioned at 5 µm. Some tissues were cryoprotected and cryosectioned at a thickness of 12 µm for TG and 30 µm for the decalcified maxillae. Immunohistochemical assays were performed using rat anti-substance P (Sigma-Aldrich, #MAB356), rabbit anti-TRPV1 (kindly provided by Dr. Michael Caterina at Johns Hopkins University), anti-CGRP (Millipore, #C7113, rabbit or Penninsula lab, guinea pig). pan-cytokeratin (BLD, #914204; mouse), and rabbit anti-CD45 (abcam, #ab10558; rabbit). The specificity of the anti-TRPV1 and anti-CGRP was previously verified (24, 25). SP primary antibody was validated by using *Tac1* ^-/-^ mice. The sections were further incubated with appropriate secondary antibodies for two hours at room temperature. Tooth sections were stained with 4’,6-diamidino-2-phenylindole (DAPI) to visualize the cellular nuclei.

### 2.5 Tartrate-resistant acid phosphatase (TRAP) staining

Maxillae were fixed overnight with 4% paraformaldehyde at 4°C and decalcified in 0.5M ethylenediaminetetraacetic acid (EDTA) for two weeks at room temperature. The decalcified tissues were dehydrated, embedded in paraffin, and sectioned into 5 µm slices. TRAP and alkaline phosphatase (ALP) staining was conducted using a commercial kit (#29467001; Fujifilm Wako Pure Chemical Corporation, Richmond, VA, USA), following the manufacturer’s protocol, with some modifications. Briefly, deparaffinized and rehydrated sections were incubated in acetate-tartaric acid buffer containing naphthol ASMX phosphate and Fast Red TR (TRAP substrate). The staining process was monitored until the color reaction became distinct. After washing, tissues were counterstained with the nuclear staining solution. In an experiment, ALP staining was performed together with TRAP staining, but it was not quantified. Digital images were scanned using an Aperio scanning system (Leica, Wetzlar, Germany). To quantify the numbers of osteoclasts in the interradicular alveolar bone surface, we selected images showing the tooth roots of both the first and second molars and the second and third molars. Dense, purple-colored, TRAP-stained multinucleated osteoclasts on the surface of bone between the roots from two teeth were counted. The number of osteoclasts from each region was normalized to the perimeter of the bone surface assessed, to calculate the number of osteoclasts per millimeter (TRAP+ cells/mm) of each sample.

### 2.6 Retrograde labeling of periodontal afferents

In C57BL/6 mice anesthetized with ketamine/xylazine, fluoro-gold (FG) (Fluorochrome LLC, Denver, CO, USA) was injected into the gingiva around the maxillary first molar to retrogradely label the periodontal afferents in the TG as described previously (23). FG was dissolved in 0.9% saline at a concentration of 4%. A 50 μl Hamilton syringe was used to slowly inject 5 μl of tracer into five sites (1 μl per site) on the gingiva around the distobuccal groove, buccal groove, mesial groove, palatal groove, and the distopalatal groove of the first maxillary molar. On seven days after injection, the ligature was placed around the maxillary first molar. The unligatured side was used as control. After two weeks, the mice were euthanized by transcardial perfusion for further histological study. After performing IHC, the FG signal was identified under a gold filter (11006v2, Chroma, Bellows Falls, VT, USA). We used Image J to calculate the surface area of the cells. We followed the criteria for neuronal size classification (small, < 300 μm^2^; medium, 300 to 600 μm^2^; large, > 600 μm^2^) (22, 26).

### 2.7 Flow cytometry

Mice were anesthetized using ketamine/xylazine and transcardial perfusion was performed using >20 ml PBS to flush out the immune cells from the vasculature. To dissect out the gingival blocks, vertical incisions were made immediately anterior to the maxillary M1 and posterior to the M3, and horizontal incisions were made at the border of the gingiva on both the buccal and palatal sides. Gingival tissues from the unligatured side were used as controls. The gingival tissues were processed as previously described (4, 27). The gingival tissues were digested in a mixture of 3.2 mg/ml type IV collagenase (Invitrogen, 17104019) and 0.15 μg/ml DNase (Sigma, DN25) in media for 25 min at 37°C with gentle shaking. Then EDTA was added to a final concentration of 2 mM, and the solution was incubated for 5 min. The enzymes were prepared in a complete media containing RPMI with 2 mM L-glutamine, 100 units/ml penicillin, 100 µg/ml streptomycin, and 10% fetal bovine serum. The enzyme-digested gingival tissues and media were mashed through a cell strainer with a pore size of 70 μm, and additional cold DNase media was added. The cell suspension was centrifuged at 4°C, at 314×g for 6 min, and the pellet was resuspended in 100 µl cold PBS containing 0.5% FBS. Gingival tissues and single cell suspensions obtained from two separate mice in the same experimental group were pooled for one flow cytometry experiment. Single cell suspensions obtained from two separate mice in the same experimental group were pooled for one flow cytometry experiment. CD45-PE, CD11b-BV421, and Ly6G-PE-Cy7 antibodies were used to identify neutrophils. Flow cytometry analysis was performed within 2 hours on Cytek Aurora flow cytometer (3 lasers: 405nm, 488nm, 640nm; Cytek Biosciences, San Jose, CA, USA). The spectral data were unmixed based on single color compensation beads controls and analyzed using FCS Express 7 software (*De Novo* Software, Pasadena, CA, USA). Neutrophils were defined by 7-AAD^-^/CD45^+^/Ly6G^+^/CD11b^+^. Percentages of each cell population in live, single cells were calculated for comparisons between groups.

### 2.8 Gingival injection of the drugs

Gingival injections of drugs were performed using an insulin syringe with a 31G needle under isoflurane anesthesia. SP (Sigma-Aldrich, # S6883; 1 µg/site), QWF (Tocris, #6645; 2 µg/site), neurokinin A (NKA; MilliporeSigma, # 86933746; 1 µg/site) or vehicles (PBS or dimethyl sulfoxide) were injected into the proximal and distal areas of the gingiva around M2.

### 2.9 Luminex multiplex assay

The mice were euthanized following anesthesia with ketamine/xylazine, and maxillae, including the gingiva, alveolar bone, and three molars, were dissected out. Whole tissues were ground in a buffer (210 µl; 50 mM Tris, 2 mM EDTA pH 7.5) containing a protease/phosphatase inhibitor cocktail (5872S; Cell Signaling Technology, Danvers, MA, USA) using a tissue grinder (Pyrex PTFE Pestle Tissue Grinder with Steel Shaft). After spinning down for 5 min at 1000 g at 4°C, the supernatant was collected and frozen on dry ice. Luminex multiplex cytokine assays were performed using Mouse Luminex Discovery Assay Kits (R&D Systems, Minneapolis, MN, USA) and a Luminex 100 Multi-analyte System (Luminex, Chicago, IL, USA).

### 2.10 Quantification and statistical analysis

All data are presented as mean ± SEM. The data were analyzed using Student’s t-tests, a one-way ANOVA, and a two-way ANOVA followed by *post hoc* assays, as indicated in the figure legends, using GraphPad Prism 7 (GraphPad Software, San Diego, California USA). Comparisons of distributions of neuronal sizes between two groups were performed using Mann-Whitney test. A value of P< 0.05 was considered statistically significant.

## 3 Results

### 3.1 *Tac1* knockout mice show reduced bone loss in periodontitis

**Figure 1:**
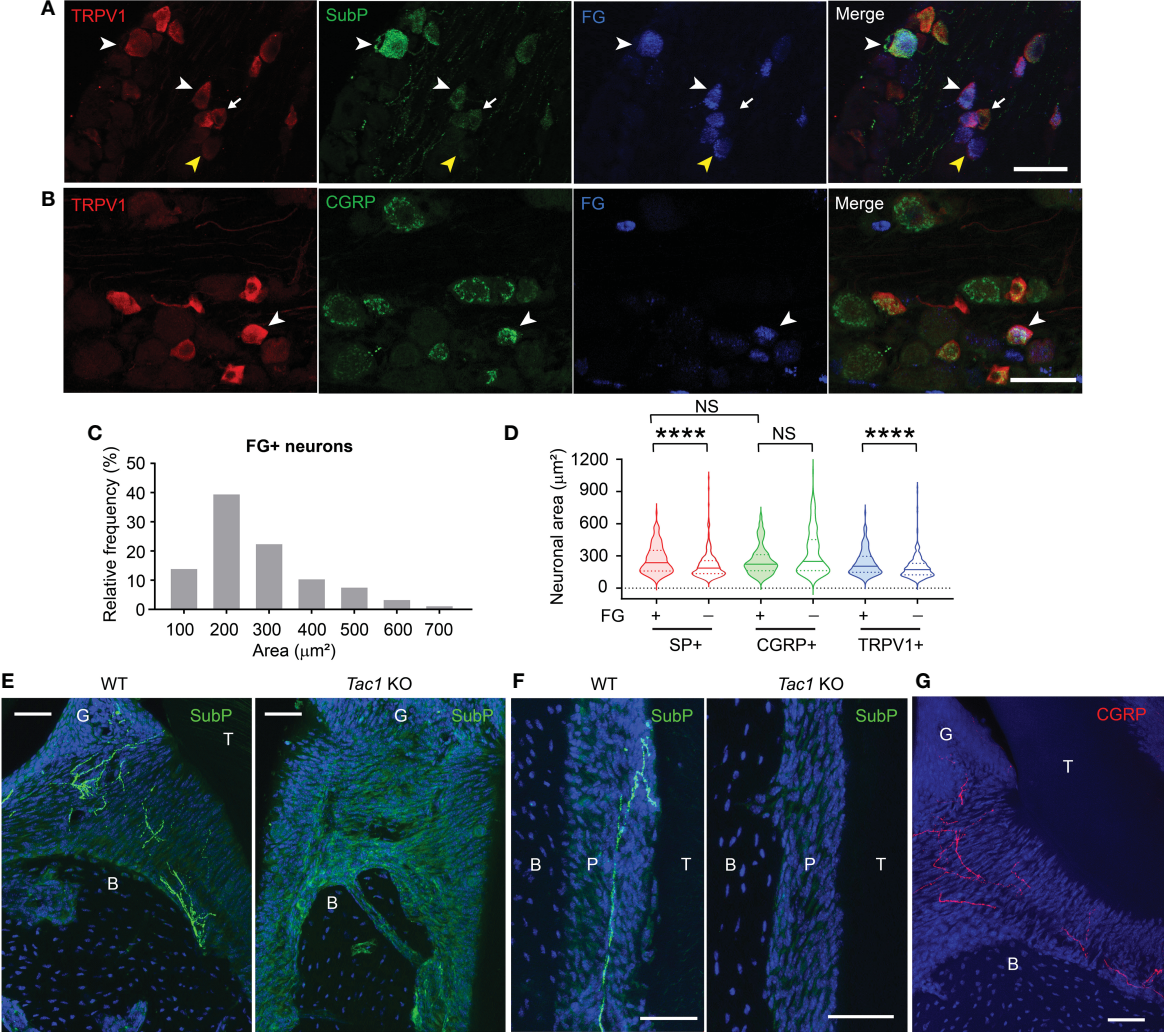
Substance P and CGRP is expressed in small to medium-sized gingival afferents. **(A)** Representative images of immunohistochemical labeling of TRPV1 (red) and substance P (SubP; green) in trigeminal ganglia (TG). Periodontal afferents were labeled by Fluoro-gold (FG), a retrograde tracer, injected into the gingiva around the maxillary second molar. White arrowheads indicate examples of FG+/TRPV1+/SP+ neurons in TG sections. The yellow arrowhead indicates an example of FG+/TRPV1+/SP-negative neuron. The arrow indicates FG-negative TRPV1+/SP+ neuron. Scale bar, 50 µm. **(B)** Representative images of immunohistochemical labeling of TRPV1 (red) and CGRP (green) in TG retrogradely labeled by FG from gingiva. The white arrowhead indicates an example of FG+/TRPV1+/CGRP+ neuron. Scale bar, 50 µm. **(C)** Size distribution of FG-labeled gingival afferents (1,234 neurons from five TG). **(D)** Violin plots comparing the size distribution of FG+ or FG-negative SP+ (red), CGRP+ (green), or TRPV1+ (blue) neurons. N=159, 231, 75, 219, 147, and 257 neurons. ****p<0.0001 in Mann-Whitney test. NS, not significant. **(E–G)** Immunohistochemical labeling of SP (green; **E, F**), CGRP (red; **G**) and DAPI (blue) in decalcified periodontia of *Tac1* KO or WT mice **(E, F)** or C57bl/6 **(G)** mice. B, bone; G, gingiva; P, periodontal ligament; T, tooth. Scale bar, 50 µm. NS, not significant.

A subset of TG neurons retrogradely labeled from gingiva expressed SP, CGRP, and TRPV1 (**Figures 1A, B**). The FG-labeled gingival TG neurons were largely small- (52.4%) or medium- (42.2%) sized neurons with only a few large-sized neurons (5.4%) (**Figure 1C**). We previously showed that 23% of TG neurons retrogradely labeled from gingiva expressed CGRP, which is highly co-expressed with TRPV1 (23). Approximately 15% of the FG-labeled neurons express SP or TRPV1, and approximately 10% of the labeled neurons co-express SP and TRPV1. The majority of SP+, CGRP+, or TRPV1+ neurons were small-sized neurons (**Figure 1D**). FG+/SP+ neurons were significantly larger than FG-negative SP+ neurons (red). FG+/TRPV1+ neurons were also significantly larger than FG-negative TRPV1+ neurons (blue). In contrast, FG+/CGRP+ neurons were not significantly different from FG-negative CGRP+ neurons (green). However, the size distribution of FG+/SP+ and FG+/CGRP+ neurons were not significantly different. SP+ nociceptive afferents abundantly project to the gingiva and periodontal ligaments (**Figures 1E, F**). In contrast, SP was undetectable in *Tac1* KO mice, confirming SP deficiency in this line. CGRP+ nociceptive terminals were also detected from periodontia (**Figure 1G**). These data suggest that SP+ or CGRP+ afferents represent a subpopulation of sensory neurons innervating periodontal tissues.

**Figure 2:**
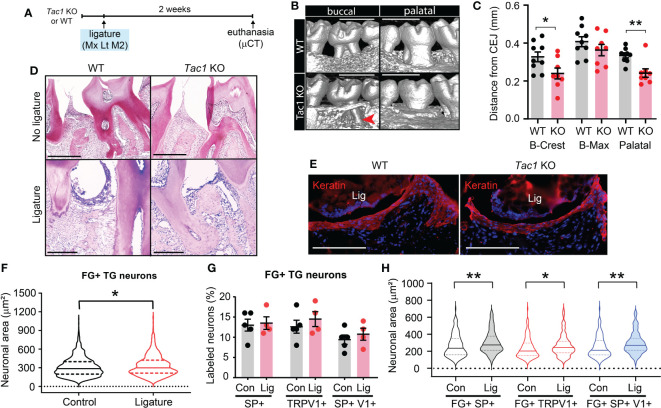
Knockout of *Tac1* reduces ligature-induced bone loss. **(A)** Time course of the experiments. Mx, Maxilla; Lt, left side ligature. **(B)** µCT examination of the effect of *Tac1* KO on bone loss two weeks after ligature placement. Arrowhead, fenestration of buccal alveolar bone on the distobuccal root of M2. Scale bar, 1 mm. **(C)** Quantification of bone loss. *p < 0.05, **p<0.01 in Student’s t-tests. N=9 in WT and 8 in KO. **(D)** H&E staining of decalcified periodontia of WT and *Tac1* KO with (bottom) or without ligature (top). Scale bar, 200 µm. **(E)** Immunohistochemical labeling of pan-cytokeratin (red) in WT and *Tac1* KO 5 days after ligature. Blue, DAPI. Scale bar, 200 µm. Lig, ligature. **(F)** Violin plots comparing the frequency distribution of FG+ neuronal areas in TG from C57bl/6 mice in control and ligature side (N=1,234 neurons from five TG in control; N=779 from four TG in ligature group). Solid line within the plot, median; dotted lines, quartiles. *p<0.05 in Mann-Whitney test. **(G)** Proportions of SP+, TRPV1+, or SP+/TRPV1+ neurons among FG+ TG neurons in the control (Con) and the ligature (Lig) group. Each point indicates a proportion in a ganglion. N=5 in the control and 4 in the ligature group. 151 to 428 FG-labeled neurons per ganglia were quantified. **(H)** Violin plots comparing the size distribution of FG+/SP+ (black), FG+/TRPV1+ (red), or FG+/SP+/TRPV1+ (blue) neurons in the control (Con) or the ligature (Lig) group (N=159, 106, 147, 118, 111, and 85 neurons). Solid line within the plot, median; dotted lines, quartiles. * p<0.05; **p<0.01 in Mann-Whitney test.

To determine functional roles of SP and CGRP, encoded by *Tac1* and *Calca* gene respectively, we used *Tac1* and *Calca* knockout mice for ligature-induced periodontitis experiments. *Tac1* knockout (KO) mice (*Tac1^-/-^*) were viable and exhibited a hypoalgesia phenotype (28). We first examined ligature-induced bone loss in the *Tac1* KO mice *via* µCT. Two weeks after placing the ligature, wild type (WT) mice showed remarkable alveolar bone loss in both the buccal and palatal sides as indicated by a decrease in alveolar bone height, as well as buccal bone plate disruption (**Figures 2A–C**). In contrast, *Tac1* KO mice exhibited an obviously reduced loss of the alveolar bones compared to WT controls. Specifically, alveolar bone height reduction and buccal bone disruption were less severe, and the buccal bone plate showed fenestration rather than complete resorption on the crest (**Figures 2B, C**). Consequently, the distance from CEJ to buccal alveolar bone crest (B-Crest) was significantly less in *Tac1* KO than WT, although the distance from CEJ to the most apical site of bone destruction (B-max) was not significantly different. Bone loss in palatal side was also significantly less in *Tac1* KO than WT. In histological sections, bone loss was more prominent in WT than in *Tac1* KO after ligature, whereas alveolar bone without ligature was comparable (**Figure 2D**). Different extent of bone loss between genotypes are unlikely due to the different extent of mucosal damage since cytokeratin-expressing gingival epithelia were comparable (**Figure 2E**).

We assessed if ligature-induced periodontitis produces changes in SP or TRPV1 expression in TG. The retrogradely labeled FG+ TG neurons from mice with ligature showed a slight but significant increase in neuronal sizes compared to controls (**Figure 2F**). The proportions of FG+/SP+ neurons, FG+/TRPV1+ neurons, or FG+/SP+/TRPV1+ neurons were not significantly different between the control and the ligature group (**Figure 2G**), suggesting that altered expressions of SP or TRPV1 in gingival afferents are not major contributors to the nociceptor regulation of periodontitis. Interestingly, the size distributions of FG+/SP+, FG+/TRPV1+, or FG+/SP+/TRPV1+ neurons were significantly larger in the ligature group than in controls, although the extent of such changes was modest (**Figure 2H**).

### 3.2 *Calca* deletion shows a marginal role in bone loss in periodontitis

**Figure 3:**
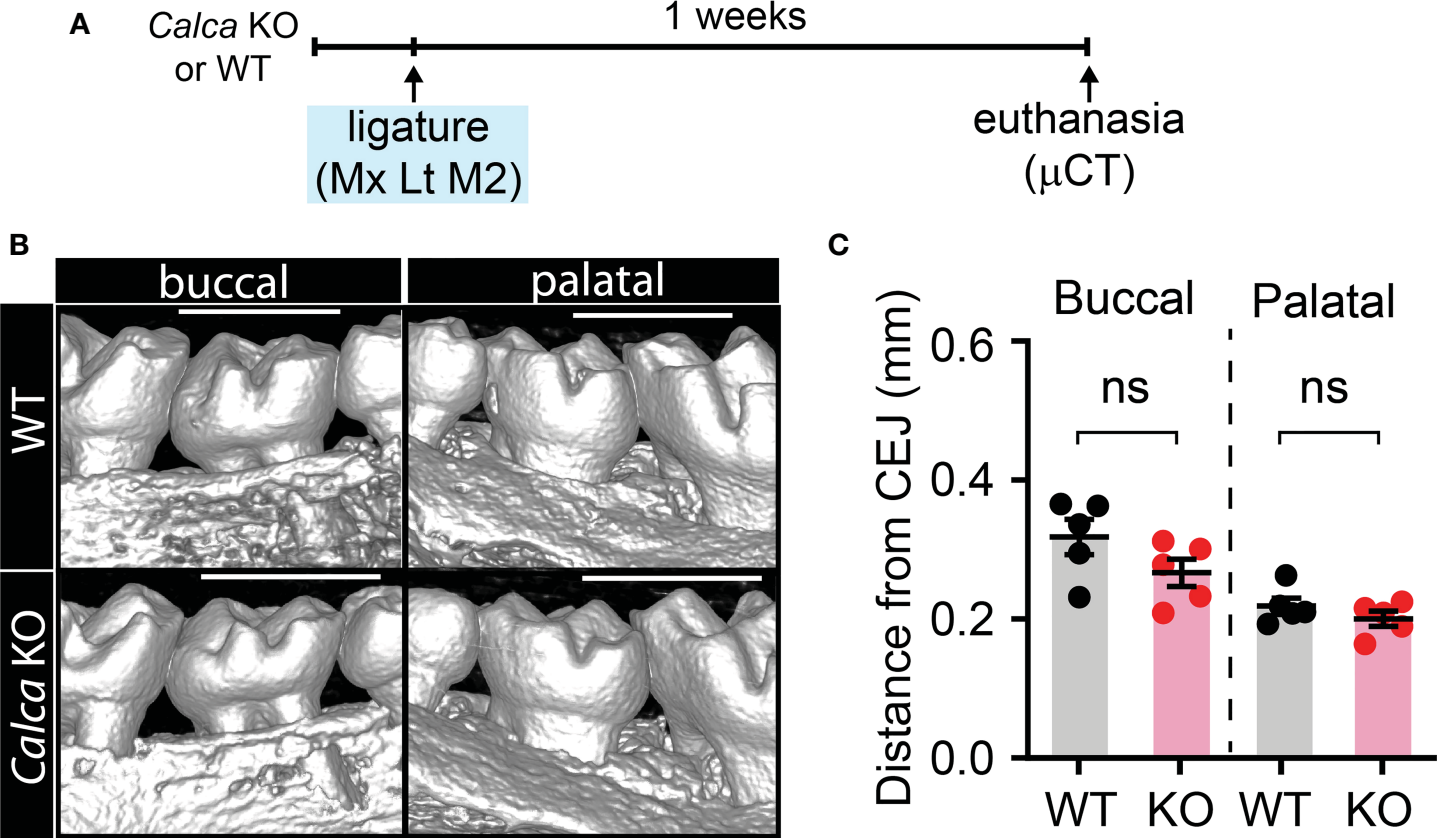
Knockout of *Calca* does not affect ligature-induced bone loss. **(A)** Time course of the experiments. **(B)** µCT analysis of bone loss one week after ligature placement in *Calca* ^Cre/Cre^, in which Cre is knocked into the locus of *Calca* without expression of *Calca*. Scale bar, 1 mm. **(C)** Quantification of bone loss. N=5 per group. ns, not significant.

Since CGRP is another type of neuropeptide abundantly expressed in nociceptors, we therefore also examined the involvement of CGRP in ligature-induced bone loss by using *Calca* KO mice that are deficient of CGRP (**Figure 3**). We found that ligature-induced bone loss in *Calca* KO mice was comparable to WT, suggesting that net effects of global knockout of CGRP may not significantly impact bone resorption in periodontitis.

### 3.3 *Tac1*-encoded neuropeptides modulate osteoclast activation and host immune responses in periodontitis

**Figure 4:**
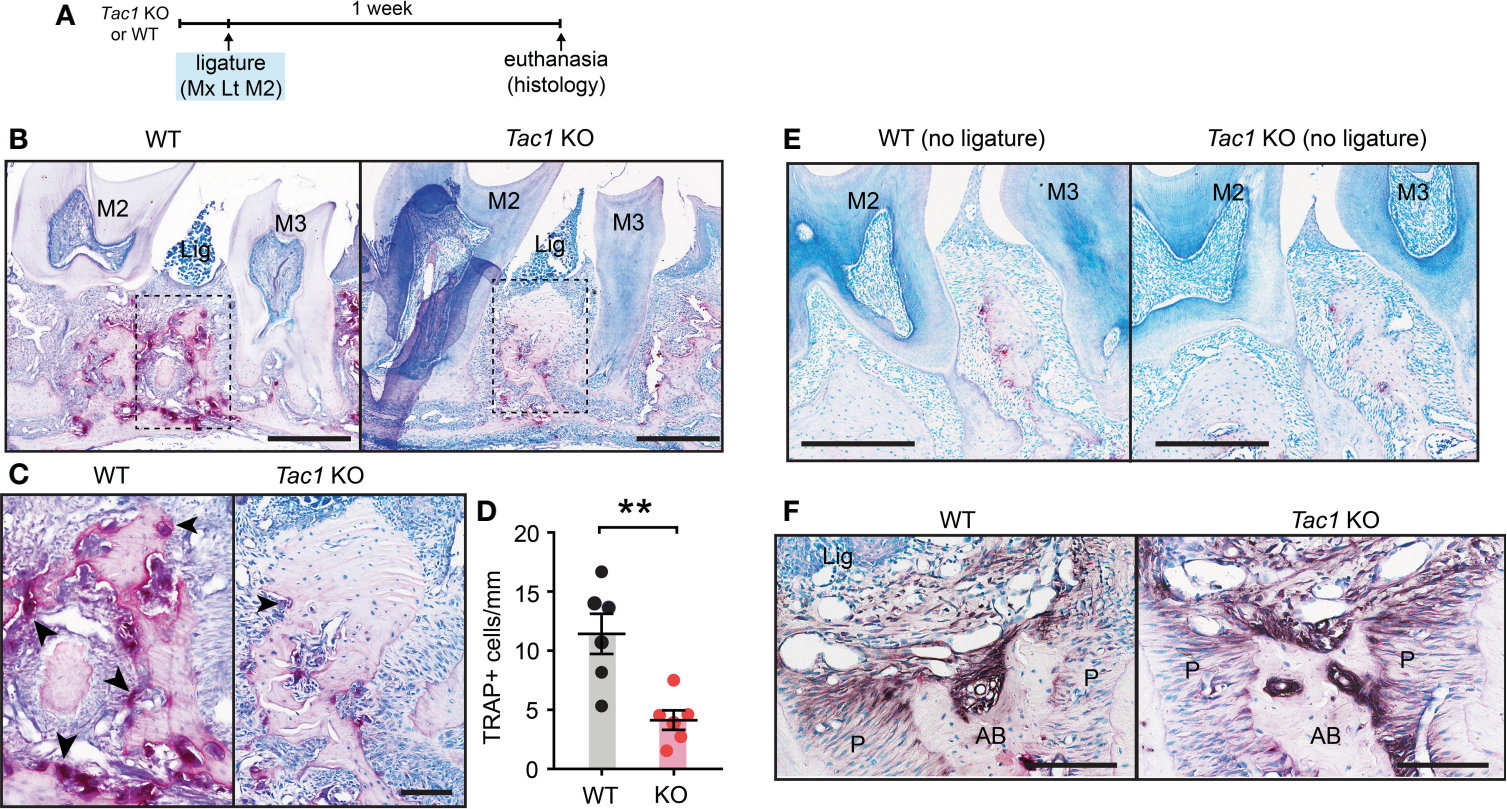
Knockout of *Tac1* reduces ligature-induced activation of osteoclasts. **(A)** Time course of the experiments. **(B)** TRAP staining five days after ligature placement in *Tac1* KO and WT mice. Lig, ligature; M2, second molar; M3, third molar. Scale bar, 500µm. **(C)** Magnified view of the insets in **(A)** Scale bar, 100µm. **(D)** Quantification of TRAP+ cells. **p < 0.01 in Student’s t-tests. N=6 per group. **(E)** TRAP staining of periodontia without ligature in WT and *Tac1 KO* mice. Scale bar, 200µm. **(F)** Alkaline phosphatase staining in *Tac1* KO and WT mice. Scale bar, 100µm. AB, alveolar bone; Lig, ligature; P, periodontal ligament.

In agreement with reduced bone loss, TRAP staining revealed that osteoclast numbers outlining the bone surfaces were also decreased in *Tac1* KO mice in comparison to controls. These histological studies were conducted five days after ligature placement, when osteoclasts and bone resorption were more actively ongoing than at the two-week time point (**Figures 4A–D**). Osteoclasts activities were comparable between genotypes without ligature (**Figure 4E**). In ligature groups, ALP activities were observed along the bone surfaces in both *Tac1* KO and WT mice in comparable extents, suggesting that *Tac1* KO did not substantially affect the bone formation process (**Figure 4F**).

**Figure 5:**
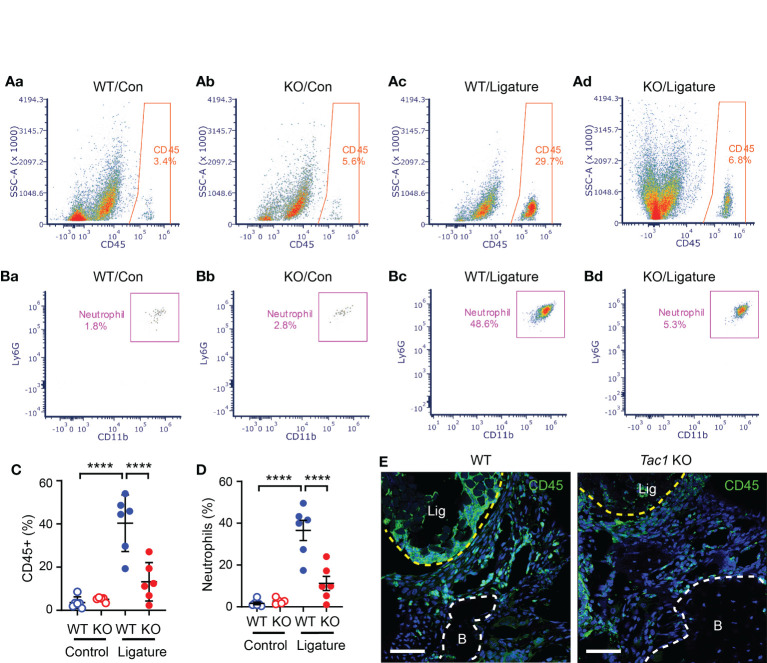
SP knockout reduces ligature-induced host responses at the site of periodontitis. **(A, B)** Flow cytometry was performed to identify the proportion of immune cells in single-cell suspensions from gingiva in control (Con; a-b) or ligature side (Ligature; c-d) in WT or *Tac1* KO mice two weeks after ligature placement. The percentage in each plot represents the fraction of the given cells out of live, single cells in each sample. Examples of total CD45+ leukocytes **(A)** and neutrophils **(B)** in WT/control, KO/control, WT/ligature, and KO/ligature groups are shown. **(C, D)** Proportions of CD45+ or neutrophils in live, single cells in each sample are plotted. ****p <0.0001 in Sidak *post hoc* tests following one-way ANOVA. N=6 per group. **(E)** Immunohistochemical labeling of CD45 in a periodontium under ligature (Lig; yellow dotted line) in WT or Tac1 KO mice two weeks after placing the ligature. Scale bar, 50 µm. B, alveolar bone; Lig, ligature.

**Figure 6:**
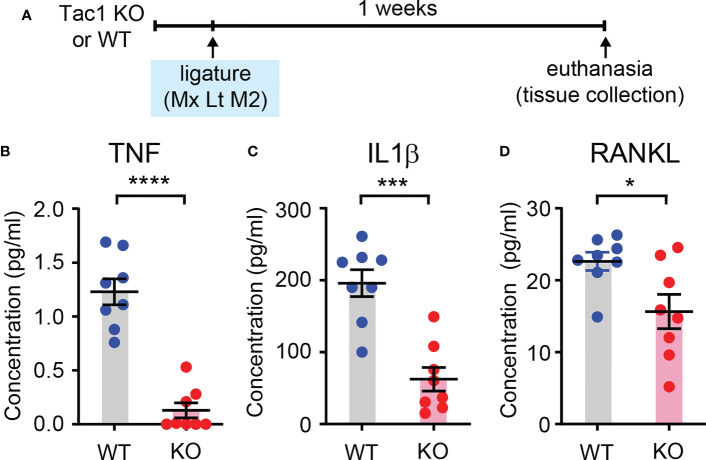
SP knockout decreases proinflammatory cytokines in periodontium. **(A)** Time course of the experiment. Luminex assay for measuring tumor necrosis factor (TNF; **B**), interleukin 1β (IL1βγ **C**) and receptor activator of nuclear factor kappa- Β ligand (RANKL; **D**) in periodontia from WT or *Tac1* KO mice. The mice were euthanized two weeks after placing the ligature. *p<0.05; ***p<0.0005; ****p<0.0001. N=8 per group.

Given that the regulation of host responses by neuropeptides underlies the neural regulation of barrier tissue functions, we further investigated the role of *Tac1* in the recruitment of immune cells to sites of periodontitis. We performed flow cytometry assays using cells collected from gingiva with and without ligature in WT and *Tac1* KO mice. The proportion of total CD45+ leukocytes (**Figure 5A**) and neutrophils (**Figure 5B**) were significantly greater in periodontia from the ligature side than from the control side in WT mice. In comparison, the proportion of ligature-induced total CD45+ leukocytes and neutrophils were significantly reduced in *Tac1* KO mice compared to WT (**Figures 5C, D**). Consistently, immunohistochemical labeling of CD45 in periodontia with ligatures also demonstrated limited recruitment of CD45+ cells in *Tac1* KO mice relative to controls (**Figure 5**). Furthermore, cytokines associated with inflammatory responses, such as tumor necrosis factor, interleukin 1β, and receptor activator of nuclear factor kappa- Β ligand (RANKL), were significantly lower in the periodontia of *Tac1* KO mice than WT (**Figure 6**). Altogether, these results indicate that *Tac1* KO mice exhibit reduced host response in ligature-induced periodontitis and suggest that SP is a major neurogenic regulator of host immune responses and alveolar bone loss in periodontitis.

### 3.4 Exogenous SP, but not NKA, induces inflammatory responses and promotes ligature-induced bone loss

**Figure 7:**
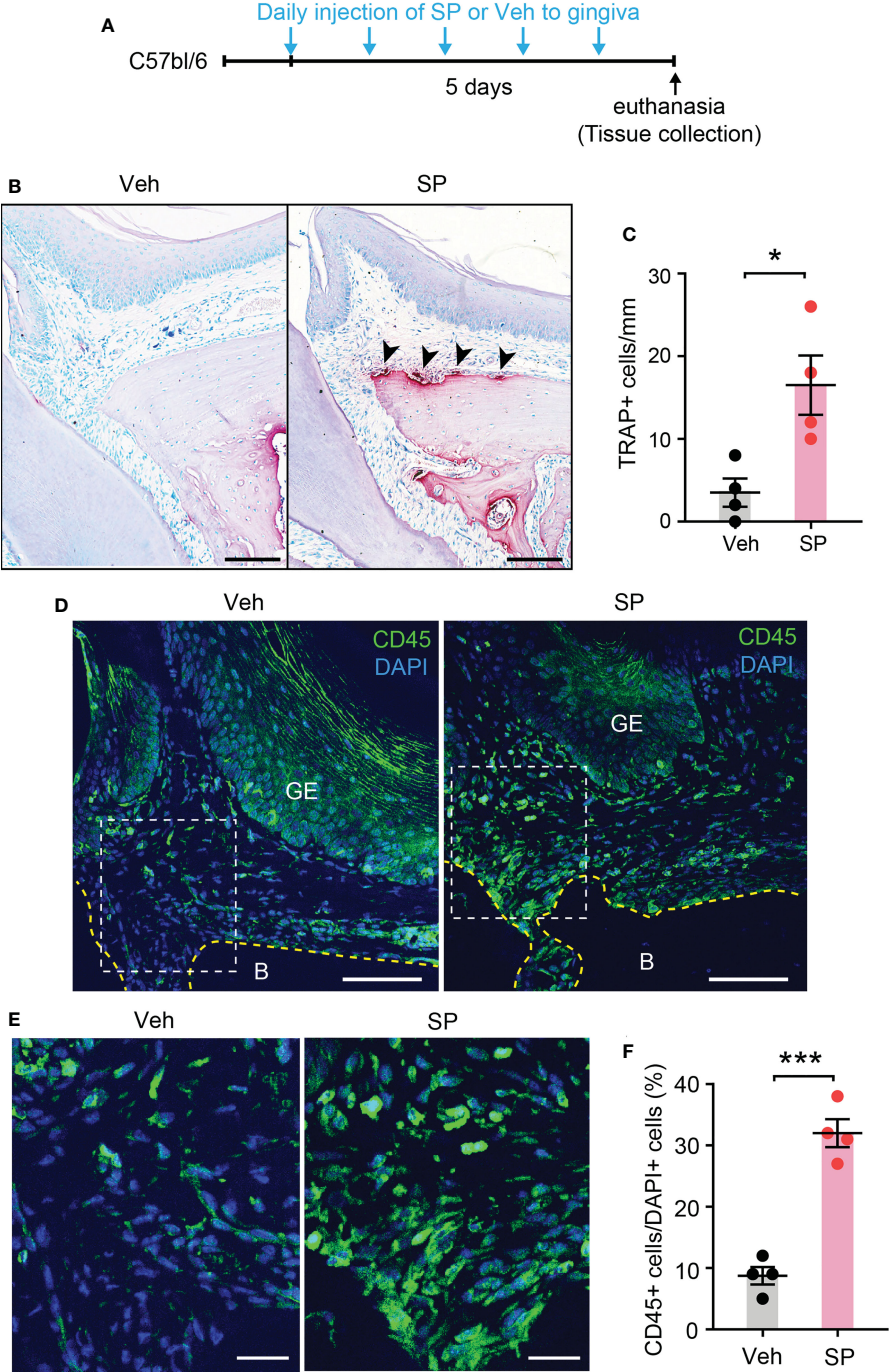
Exogenous injection of SP is sufficient to activate osteoclasts and to recruit immune cells in periodontium. **(A)** Time course of the experiment. **(B)** TRAP staining in substance P (SP; 1 µg/site)- or vehicle (Veh) injected into a periodontium. Scale bar, 100 µm. **(C)** Quantification of TRAP+ cells of the injected periodontia. *, p<0.05 in Student’s t-test. N=4 mice per group. **(D)** Immunofluorescence for CD45 in a periodontium injected with Veh or SP. Scale bar, 100µm. B, bone; GE, gingival epithelium. **(E)** Magnified view of the insets in panel **(D)** Scale bar, 30µm. **(F)** Quantification of CD45+ cells. Percentage of the number of CD45+ cells among DAPI+ cells in gingival epithelium and connective tissues within 600 µm distance to tooth surface was calculated. ***p<0.0001 in Student’s t-test. N=4 mice per group.

**Figure 8:**
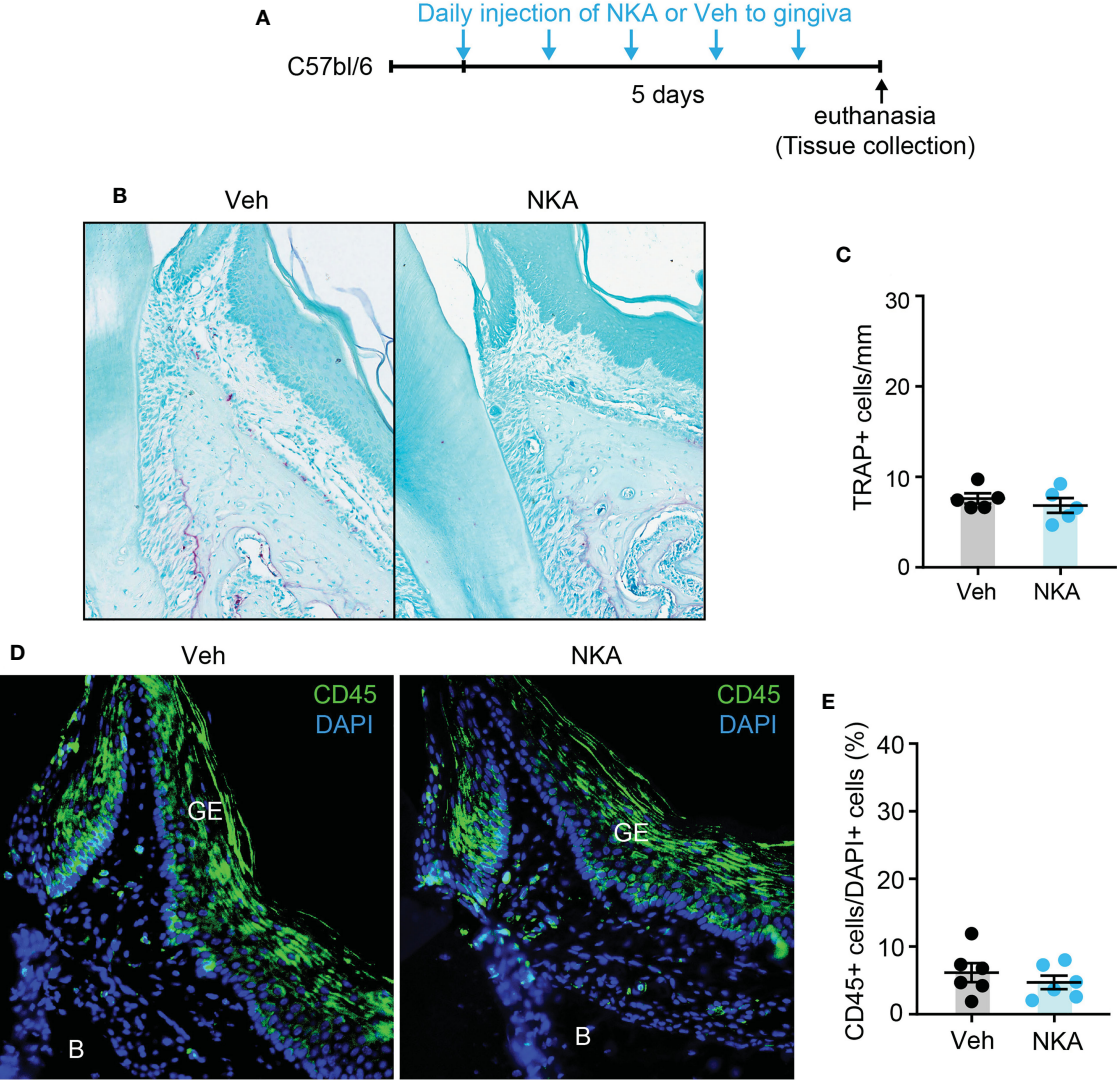
Exogenous injection of neurokinin A is not sufficient to activate osteoclasts and to recruit immune cells in periodontium. **(A)** Time course of the experiment. **(B)** TRAP staining in neurokinin A (NKA; 1 µg/site)- or vehicle (Veh) injected into a periodontium. Scale bar, 100 µm. **(C)** Quantification of TRAP+ cells of the injected periodontia. N=6 mice per group. P>0.4 in Student’s t-test. N=6 mice per group. **(D)** Immunofluorescence for CD45 in periodontia injected with Veh or NKA. Scale bar, 100µm. B, bone; GE, gingival epithelium. **(E)** Quantification of CD45+ cells. Percentage of the number of CD45+ cells among DAPI+ cells in gingival epithelium and connective tissues within 600 µm distance to tooth surface was calculated. P>0.7 in Student’s t-test. N=5 mice per group.

Since *Tac1* encodes both SP and NKA and both are detected in human gingiva crevicular fluid (17, 29), we determined if local injection of exogenous SP or NKA into gingiva is sufficient to induce osteoclastic activities and gingival inflammation. Results showed that injections of SP alone without periodontal ligature increased the number of osteoclasts along the bone surfaces of the injected areas (**Figures 7A–C**). Moreover, infiltration of CD45+ cells was also increased in SP-injected areas compared to vehicle-injected areas (**Figures 7D–F**). In contrast, the injection of exogenous NKA did not induce the osteoclast activity (**Figures 8A–C**) or CD45+ cell infiltration (**Figures 8D, E**). These results support the idea that Tac1 KO phenotypes inducing the inflammatory responses and osteoclasts activation are dominantly mediated by SP, but not by NKA.

**Figure 9:**
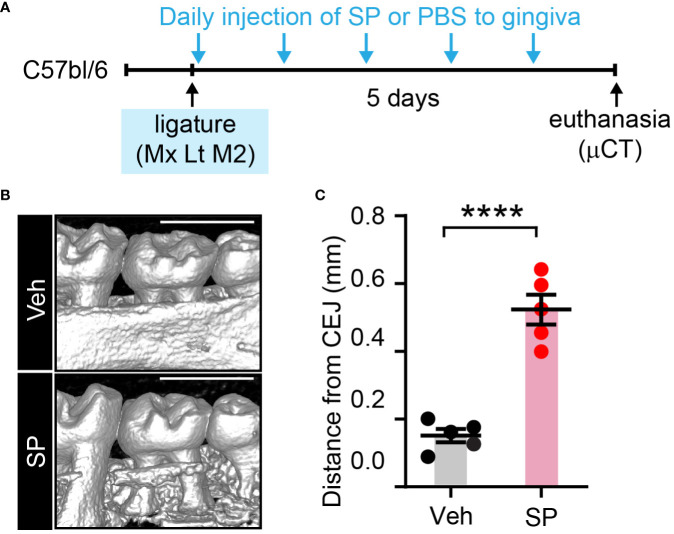
Exogenous substance P aggravates ligature-induced periodontitis. **(A)** Time course of the experiment. Under isoflurane anesthesia, SP (1 µg/site) or vehicle (PBS) was injected twice a day into two sites in the gingiva around the maxillary second molar; one site between the first and second molars, and the other site between the second and third molars) for five days after placing the ligature. **(B)** µCT examination of a periodontium five days after ligature placement with Veh or SP injection. Scale bar, 1 mm. **(C)** Quantification of bone height loss. ****p<0.0001 in Student’s t-test. N=5 per group. CEJ, cement-enamel junction.

To further test the roles of exogenous SP in periodontitis, we examined ligature-induced bone loss in mice with local injections of SP or vehicle (**Figure 9**). Consistently, the SP-injected group demonstrated more vigorous bone resorption compared to the Veh-injected group.

### 3.5 Pharmacological inhibition of SP receptors decreases ligature-induced bone loss resembling *Tac1* KO mice

**Figure 10:**
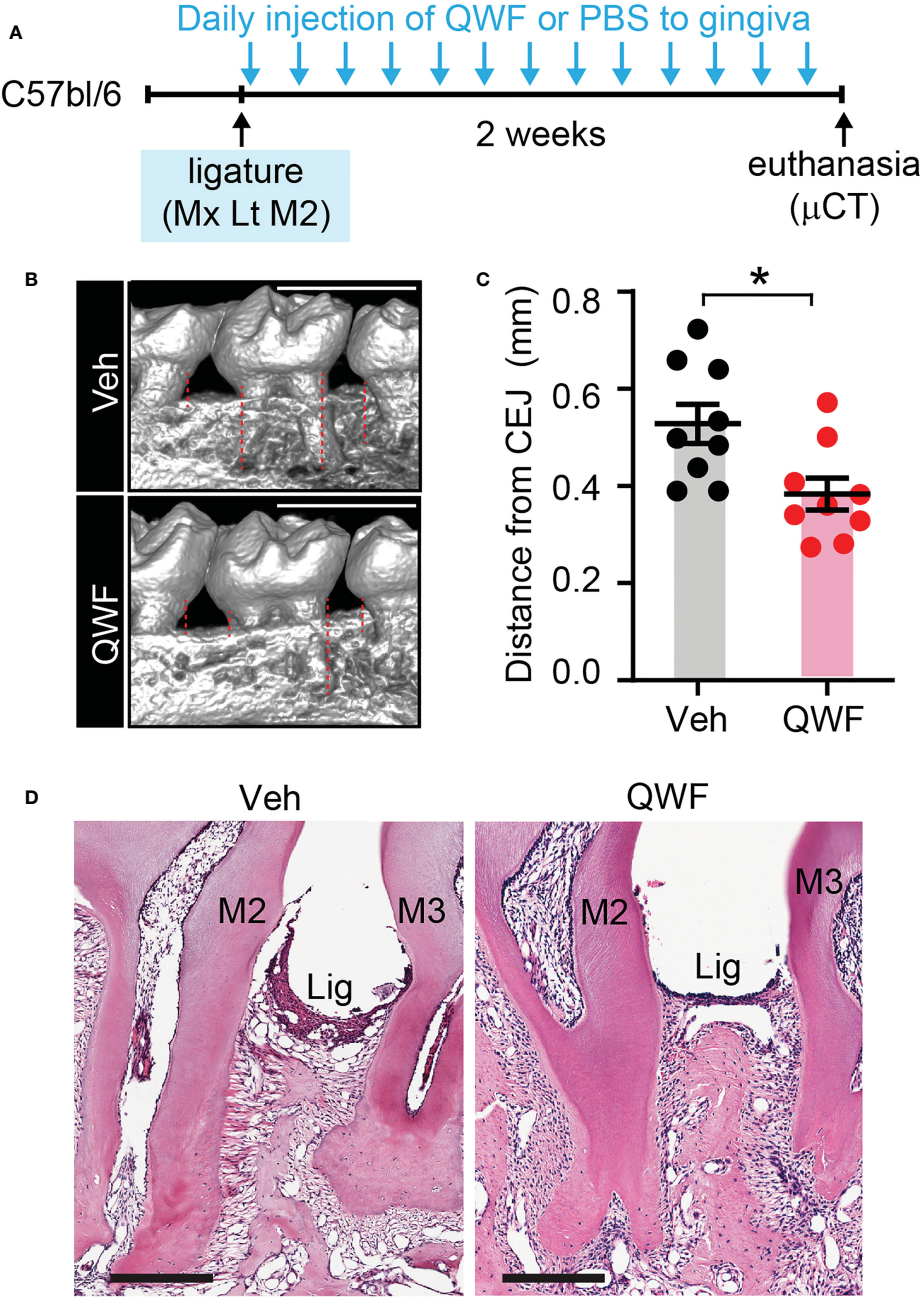
Substance P receptor antagonist reduces ligature-induced periodontitis. **(A)** Time course of the experiment. Under isoflurane anesthesia, QWF (2 µg/site) or vehicle (PBS) was injected twice a day into two sites in the gingiva around the maxillary second molar; one site between the first and second molars, and the other site between the second and third molars) for 14 days after placing the ligature. **(B)** µCT examination of a periodontium with vehicle or QWF injection. Scale bar, 1 mm. Dotted lines represent the measurements. **(C)** Quantification of bone loss. *p<0.05 in Student’s t-test. **(D)** Representative images of H&E stained sagital sections. N=8 per group.

We also determined the effects of inhibiting SP receptors in the periodontium by performing gingival injections of QWF, a tripeptide SP antagonist, or vehicle with periodontal ligature (**Figures 10A–D**). Results showed that QWF injection significantly decreased bone loss compared to the vehicle injection, which is consistent with the effects of *Tac1* KO.

## 4 Discussion

It has been suggested that there are neural regulatory mechanisms for bone homeostasis, with sensory neurons playing an important role in regulating bone metabolism and remodeling (30, 31). Dysregulation of neural activity is involved in a number of pathological conditions affecting the skeletons. Recently, we showed that chemical ablation of nociceptors by resiniferatoxin (RTX) or chemogenetic silencing of TRPV1-lineage afferents reduced bone loss in a periodontitis mouse model (4). Here, we have further demonstrated that periodontal ligature applied to *Tac1* KO mice reduces alveolar bone loss, activation of osteoclasts, the recruitment of inflammatory cells to the periodontium, and cytokine levels. These results fully recapitulate the phenotype of mice with RTX induced-ablation or chemogenetic silencing of nociceptors. Moreover, we have shown that SP injections alone are sufficient to induce robust inflammation and bone resorption in the periodontium, while local pharmacological inhibition of SP receptors in gingiva decreases bone loss. Altogether, our data indicate that nociceptor Mediated SP signaling plays an important role in regulating bone resorption in periodontitis. In contrast, while CGRP has been suggested as an anabolic regulator of alveolar bone loss through *in vitro* assays (7), we found that *Calca* KO mice did not show significant differences in alveolar bone loss *in vivo*. We assume that CGRP-mediated anabolic effects are overwhelmed by the catabolic effects of SP, and the simultaneous release of SP and CGRP from nociceptor terminals induces a net increase in bone loss.

Retrograde labeling of gingival afferents showed that >90% of gingival afferents are small to medium-sized neurons. This is in contrast with pulpal afferents, in which large-sized neurons are highly enriched (49%) (22). Our previous (23) and the current study showed that 23% and 15% of gingival afferents express CGRP and SP, respectively. Therefore, it would be reasonable to estimate that approximately a quarter of gingival afferents are peptidergic afferents. The proportion of the SP+ or TRPV1+ gingival afferents was not changed after placing the ligature. Inflammation of masseter muscle produced by complete Freund’s adjuvant upregulates the expression of SP and TRPV1 in TG whereas orthodontic tooth movement of the maxillary first molar does not induce such changes (32, 33). Therefore, upregulation of SP and TRPV1 in TG may be context dependent and the extent of tissue inflammation produced by ligature is not as strong as masseter inflammation. The analysis of size distribution showed that SP+ and TRPV1+ gingival afferents were larger than SP+ and TRPV1+ non-gingival (unlabeled) TG neurons, whereas CGRP+ gingival afferents were not different from CGRP+ non-gingival afferents. The size distributions of SP+ and CGRP+ peptidergic afferents were not significantly different. Comparing the size distributions between the control and the ligature group, SP+ or TRPV1+ gingival afferents were modestly shifted toward a larger range in the ligature group. These results suggest that gingival SP+ and TRPV1+, but not CGRP+, afferents are uniquely larger than non-gingival afferents, and the placement of ligature may upregulate SP and TRPV1 in medium-sized neurons. Alternatively, given the finding that the distribution of the entire FG+ neuronal sizes was also shifted in the ligature group, one possibility is that the harmful effects of retrograde labeling dye (e.g (34),) lead to the degeneration of a subset of small-sized TG neurons, which might be aggravated by the ligature-induced inflammation. Further studies using independent approaches (e.g., different chemical or viral tracers) may clarify it. Nonetheless, given the modest extent of changes, we presume that altered expressions of SP or TRPV1 in gingival afferents are not major contributors to the nociceptor regulation of periodontitis.

The *Tac1* gene (also known as *PPT-1* or *PPT-A*) encodes both SP and NKA (29). *Tac1* produces multiple isoforms of mRNA transcripts through alternative splicing, which further produces SP and NKA *via* post-translational modification. SP and NKA are co-synthesized and co-released from sensory neurons and are both implicated in periodontitis (14, 15). Therefore, our data using *Tac1* KO mice suggests that both SP and NKA are involved in the regulation of periodontal bone loss. Untangling the relative roles of *Tac1-*encoded SP and NKA in biological processes is challenging. As SP and NKA preferably bind to the G-protein linked NK1 receptor (NK1-R) and the NK2 receptor (NK2-R) respectively, and both receptors demonstrate broad expressions in periodontal tissues, specific inhibition of NK1-R and NK2-R may indicate the respective contributions of SP and NKA. Indeed, our data using QWF, an efficacious inhibitor of NK1-R, does suggest the dominant contribution of SP in periodontal bone loss. Furthermore, direct injection of SP, but not NKA, into gingiva was sufficient to produce immune cell recruitments and osteoclastic activation, which supports the dominant role of SP compared to NKA. These data suggest that specific NK1-R antagonists, such as FDA-approved anti-emetic Aprepitant (35), might be considered as a supplemental therapeutic approach to delay the development or progression of periodontitis.

NK1-R is broadly expressed in both immune and bone cells, and SP may, therefore, directly regulate the inflammatory process and bone remodeling under physiological and pathological conditions (36–38). SP enhances both osteogenesis and bone resorption *in vitro* (39–41). SP increases differentiation of osteoblasts and increases osteogenic activity at low concentration (39). Consistently, *Tac1* KO mice show reduced bone volume and trabecular number/thickness in femur (42). At the higher concentrations that are likely detected at the site of injury or inflammation, SP enhances differentiation and resorptive activity of osteoclasts (39, 43, 44). Our results suggest that *Tac1* deficiency does not globally influence alveolar bone without ligature but protects bone loss following ligature placement. Therefore, SP-induced osteoclast accumulation and differentiation may significantly contribute to the bone loss seen in periodontitis. In addition to NK1R, SP also binds to the mast cell surface receptor MRGPRB2 to activate mast cells, which play a significant role in the inflammatory process—including periodontitis (38, 45). Therefore, neural-mast cell interactions could lead to the regulation of the progression of periodontitis. In future studies, an analysis of the relative roles of NK1-R, NK2-R, and MRGPRB2 in periodontitis would be a rational approach to provide mechanistic insight into SP’s regulation of periodontal bone loss.

Aside from nociceptors, SP expression has also been reported in many other tissues, including inflammatory cells in periodontitis models (12, 44). However, these results should be interpreted with caution, as many SP antibodies may also detect other members of the tachykinin family (36, 46). A study using RNA *in situ* hybridization has demonstrated that SP is only expressed in a highly restricted number of cells (aside from neurons) during the inflammatory process (46). Therefore, we believe nociceptors remain the primary source of SP release in periodontitis, particularly at an early stage of inflammation.

In the current study, we did not attempt to identify the upstream signal which trigger the release of SP from afferent terminals under periodontitis. TRPA1 and TRPV1 are largely co-expressed in nociceptors and are both involved in various inflammatory processes (5). Tissue inflammation generates putative endogenous agonists of TRPV1 and TRPA1, such as byproducts of oxidative stress (47, 48). Inflammatory mediators such as prostaglandin E2 or bacterial toxins such as *P. gingivalis*-derived lipopolysaccharides can activate or sensitize TRPV1 and TRPA1 (49–51). TRPA1 activation then evokes SP secretion in dorsal root ganglion neurons (52). Therefore, it is possible that periodontitis-induced TRPV1/TRPA1 signaling triggers SP secretion from nociceptors in ligature-induced periodontists. Interestingly, however, TRPV1 KO mice have previously been reported to increase ligature-induced bone loss (7). One possibility is that the global KO of TRPV1 affects the function of osteoclasts, which may express functional TRPV1 (53, 54). Conditional KOs of TRPV1 specific to sensory neurons should reveal the relative contributions of neuronal and osteoclastic TRPV1.

Despite the substantial contributions of the peptidergic nociceptive afferents to periodontitis, it is puzzling that chronic periodontitis is not usually accompanied by pain. The molecular mechanisms of such painless periodontitis are not well understood but might involve a multitude of unique bacteria-host responses in periodontitis (55, 56). For example, mechanical hyperalgesia in gingiva does not occur by inoculating *P. gingivalis*, which is due to the inhibition of macrophages by CXCR4 in gingiva (57). Interestingly, lipopolysaccharides from *P. gingivalis* increase interleukin-10, an anti-inflammatory cytokine, upon injection into the skin (58). Further studies are warranted to determine the unique interactions of periodontal bacteria and the nociceptive system. Potential site-specific mechanisms of nociceptor regulation of local immunity and their contributions to the progression of alveolar bone pathology are also highly intriguing. While our data support the role of SP+ nociceptive afferents in aggravating marginal periodontitis, Nav1.8-expressing nociceptors, which include a substantial proportion of peptidergic nociceptors, show the opposite regulation of apical periodontitis (59). Besides nociceptive afferents, we do not exclude the potential roles of other neurochemically distinct subpopulations of gingival afferents in the alveolar bone remodeling in periodontitis. For example, glutamate is known to regulate bone homeostasis (60), and gingival afferents expressing glutamate, but not CGRP or SP, can regulate periodontal bone loss. These potential mechanisms of neural regulations of alveolar bone remodeling need to be further explored in the future.

In summary, we suggest that SP from nociceptors is a neuroimmune axis that modulates host responses and periodontitis-induced bone loss. Therefore, manipulating this axis, e.g., by localized inhibition of SP signaling in affected gums, could provide novel therapeutic approaches for treating periodontitis that supplement conventional therapies.

## Data availability statement

The original contributions presented in the study are included in the article/supplementary materials. Further inquiries can be directed to the corresponding author.

## Ethics statement

The animal study was reviewed and approved by University of Maryland Baltimore Institutional Animal Care and Use Committee.

## Author contributions

SW, YS, XN, VT-M, and M-KC conceived the study. SW, XN, VT-M, XF, YA, and M-KC designed the experiments. SW, YS, XN, YA, LP, and XF collected and analyzed the data. SW, XN, YS, VT-M, XF, YA, LP, and M-KC interpreted the results. YS, XN, and M-KC prepared the manuscript, and other authors critically read and commented on the manuscript. M-KC supervised all aspects of the project. All authors contributed to the article and approved the submitted version.
